# Electroencephalographic delta/alpha frequency activity differentiates psychotic disorders: a study of schizophrenia, bipolar disorder and methamphetamine-induced psychotic disorder

**DOI:** 10.1038/s41398-018-0105-y

**Published:** 2018-04-12

**Authors:** Fleur M Howells, Hendrik S Temmingh, Jennifer H Hsieh, Andrea V van Dijen, David S Baldwin, Dan J Stein

**Affiliations:** 1 Department of Psychiatry and Mental Health, University of Cape Town, Cape Town, South Africa; 2 Neuroscience Institute, University of Cape Town, Cape Town, South Africa; 3 Clinical and Experimental Sciences, University of Southampton, Southampton, UK; 4 MRC Unit on Risk and Resilience in Mental Disorders, Department of Psychiatry and Mental Health, University of Cape Town, Cape Town, South Africa

## Abstract

Electroencephalography (EEG) has been proposed as a neurophysiological biomarker to delineate psychotic disorders. It is known that increased delta and decreased alpha, which are apparent in psychosis, are indicative of inappropriate arousal state, which leads to reduced ability to attend to relevant information. On this premise, we investigated delta/alpha frequency activity, as this ratio of frequency activity may serve as an effective neurophysiological biomarker. The current study investigated differences in delta/alpha frequency activity, in schizophrenia (SCZ), bipolar I disorder with psychotic features and methamphetamine-induced psychosis. One hundred and nine participants, including individuals with SCZ (*n* = 28), bipolar I disorder with psychotic features (*n* = 28), methamphetamine-induced psychotic disorder (MPD) (*n* = 24) and healthy controls (CON, *n* = 29). Diagnosis was ascertained with the Structured Clinical Interview for Diagnostic and Statistical Manual of Mental Disorders, 4th Edition disorders and current medication was recorded. EEG was undertaken in three testing conditions: resting eyes open, resting eyes closed and during completion of a simple cognitive task (visual continuous performance task). EEG delta/alpha frequency activity was investigated across these conditions. First, delta/alpha frequency activity during resting eyes closed was higher in SCZ and MPD globally, when compared to CON, then lower for bipolar disorder (BPD) than MPD for right hemisphere. Second, delta/alpha frequency activity during resting eyes open was higher in SCZ, BPD and MPD for all electrodes, except left frontal, when compared to CON. Third, delta/alpha frequency activity during the cognitive task was higher in BPD and MPD for all electrodes, except left frontal, when compared to CON. Assessment of EEG delta/alpha frequency activity supports the delineation of underlying neurophysiological mechanisms present in psychotic disorders, which are likely related to dysfunctional thalamo-cortical connectivity. Delta/alpha frequency activity may provide a useful neurophysiological biomarker to delineate psychotic disorders.

## Introduction

Psychotic symptoms are characteristic of schizophrenia (SCZ), methamphetamine-induced psychotic disorder (MPD) and may also be seen in bipolar disorder (BPD). Electroencephalography (EEG) is a non-invasive method for recording electrical brain activity, providing a reliable measure of direct brain activity with higher temporal resolution than functional imaging^1,2^. EEG may therefore be a particularly useful approach in delineating neurophysiological mechanisms that underlie different psychotic disorders^1–4^. However, findings are not always consistent, and studies have largely focused on a single rather than multiple psychotic disorders^5–8^, and are often unable to address the effect of medications on frequency activity^4,9,10^.

Delta synchronisation, increased EEG delta activity, has been reported in SCZ and BPD, with an absence of EEG studies in MPD. Previous studies support greater delta synchrony during resting eyes closed (REC) for SCZ when compared with controls (CON)^11–20^. Few studies in SCZ have addressed relative delta activity during resting eyes open (REO) or during the completion of a cognitive task^21,22^. BPD has also been shown to have greater delta synchrony compared to CON during REC and REO^4,23,24^, although again not all data are consistent^25^. Delta synchronisation is strongly associated with CNS depression, as seen during slow-wave sleep, in coma and during anaesthesia, all conditions characterised by decreased levels of consciousness^26–29^. Global delta synchrony in CNS depression is suggested to result from assembly of subthreshold activity of GABAergic project neurons emanating from the thalamic reticular nucleus^30,31^. A second thalamic nucleus, the lateral geniculate nucleus, with simple attentional stimulation, for example, REO with cross-hair fixation, has been reported to reduce global delta synchrony^32^. The subthreshold activity of GABAergic thalami projection neurons may underlie the presentation of increased delta synchrony reported in SCZ and BPD.

Alpha desynchronisation, decreased alpha activity, has been reported in SCZ and BPD, but no studies are available in MPD. In SCZ alpha desynchronisation is reported in adolescent onset SCZ, first-episode SCZ and un-medicated and medicated SCZ^13,14,17,18,20,23,33–39^. In BPD, reduction of alpha activity during resting conditions is also supported^4,23,40,41^. Attenuation of alpha synchronisation has been strongly associated with CNS depression, as is seen with prolonged use of alcohol, anaesthesia and vegetative state, all conditions characterised by decreased levels of consciousness^42–44^. The presence of alpha synchronisation is indicative of healthy resting wakefulness with readiness to process salient information^45^. Two key networks underlie alpha desynchronisation. First, activation of the visual system, mediated by the reticular activating system^46,47^, as is seen from REC to REO where there is a mass desynchronisation of alpha. Second, desynchronisation of alpha activity is purported to reflect diverse changes in thalamo-cortical and cortical network communication^45,48–50^. The exaggerated desynchronisation of alpha activity in psychotic disorders has been suggested to represent inappropriate arousal and readiness to attend to information, whether internal or external^45^. We expect to see alpha desynchronisation in SCZ, BPD and MPD; however, this desynchronization may differ across three testing conditions: REC, REO and during the completion of a cognitive task.

On the premise of the current literature delta/alpha frequency activity may serve to delineate the psychotic disorders neurophysiologically. The aims of this study were to (1) extend current understanding of EEG frequency activity using delta/alpha ratio as a potential biomarker in psychosis by: (a) inclusion of participants with one of three psychotic disorders (SCZ, BPD I with history of psychosis, MPD); (b) record of delta/alpha frequency activity during three laboratory testing conditions—REO, REC and a simple cognitive task, a visual continuous performance task (CPT). Then (2) investigate differences in delta/alpha frequency activity in relation to prominent prescription medications. We hypothesised that psychotic disorders delta/alpha frequency activity would differ from demographic healthy controls and would serve to delineate the psychotic disorders, regardless of current medication regimes.

## Subjects and methods

The investigation was a case-controlled study involving recruitment of clinically stable outpatients from the Western Cape Province, South Africa. Three psychotic disorder diagnoses were included namely SCZ, bipolar I disorder with a significant history of psychosis (BPD), and MPD. CON participants were recruited from similar socio-economic backgrounds as patient participants.

The study was approved by the Health Sciences Research Ethics Committee, University of Cape Town (HREC Ref. No.: 192/2010). Western Cape Provincial and Hospital approval was also obtained. All research activities were conducted in accordance with the Declaration of Helsinki^51^. All research participants provided voluntary informed consent.

Participants visited the laboratory twice. The first visit included provision of informed consent and an assessment clinical interview, using Structured Clinical Interview for DSM (SCID-I) using the Diagnostic and Statistical Manual of Mental Disorders, 4th Edition, Text Revision (DSM-IV-TR)^52^ conditions. Control participants were excluded if there was a history of psychotic symptoms or family history of psychotic disorder. Participants with a psychotic disorder were excluded if they did not meet the diagnostic criteria for the study conditions: for example, participants with schizoaffective disorder were excluded. Participants were also excluded if they were younger than 19 years or older than 40 years, had general medical conditions that required prescription medications, had apparent learning disability, major brain trauma/surgery, any history of cardiovascular insult, individual or family history of epilepsy, medical implants or any metal within their person, for example, shrapnel. Female participants were excluded if they were pregnant or lactating. Patients with SCZ or BPD were excluded if any of their episodes were considered to be related to the use of a substance. MPD included psychotic symptoms with onset during methamphetamine intoxication or withdrawal and did not persist beyond 1 month since the last use of methamphetamine, or evidence of an underlying ‘primary’ psychotic disorder not related to the use of methamphetamine^52^. Evidence that the symptoms are better accounted for by a psychotic disorder that is not methamphetamine induced included the following: the symptoms precede the onset of the methamphetamine use; the symptoms persist for a substantial period of time (e.g. about a month^52^) after the cessation of acute withdrawal or severe intoxication, or are substantially in excess of what would be expected given the amount of methamphetamine used or the duration of use; or there is other evidence that suggests the existence of an independent non-MPD (e.g. a history of recurrent non-methamphetamine-related episodes). Patients with MPD were excluded if it was unclear if methamphetamine was causal to their symptoms or diagnosis, and if any of their psychotic episodes may have been related to another substance of abuse.

The second visit included a full morning of brain imaging. All EEGs were performed between 1100 and 1200 h, on a weekday. All clinical scales were performed on the same day and after the morning of brain imaging by trained clinical personnel.

Prior to obtaining EEG records, participants were familiarised to the different conditions: REO, REC, and a visual CPT. For REO, using E-prime^53^, a cross-hair, +, was presented on the screen and participants were asked to relax and look at the cross-hair. For REC ‘CLOSE EYES’ was presented on the screen in front of them. Records of 3 min EEG were obtained for each of the resting-state conditions. For the CPT the researcher explained the task requirements and participants completed a 30-s practice session, repeated if necessary until the participant was clear about task requirements.

The CPT included cueing of two S’s and the target a third consecutive S, that is, S-S-S or Cue-Cue-Target. Sixty trials of S-S-S were presented with 300 non-vowel inter-stimuli, no Xs or vowels were used. In addition, the presentation of 40 trick-Ss were embedded (these were single S’s). The total number of stimuli presented, including S-S-S, trick-Ss and inter-stimuli, was 540. The interval between presentation of stimuli was 100 ms, and presentation of stimuli was 500 ms, only a correct response to the target that was shorter than 500 ms resulted in shorter presentation of target S, the average duration of the task being 11 min. Behavioural data were extracted using E-prime^53^ and cross-checked with analogue inputs to EEG data file, Acknowledge 4.1 (Biopac Systems, Inc.). Behavioural data extracted included the number of correct responses, response time duration and errors of omission and commission.

EEG recording of REO, REC and CPT was undertaken using a simple EEG montage that included frontal (F_3_ and F_4_), central (C_3_ and C_4_) and parietal (P_3_ and P_4_) electrodes. Standard 10/20 caps (Electro-Cap International, Inc.) were used, of either medium or large size depending on head circumference of participant. Participants were grounded peripherally, linked earlobe reference was applied, and electrooculography (EOG) was recorded. The EEG system used was the Biopac MP150 system with 100 C EEG amplifiers and EOG amplifier (Biopac Systems, Inc.). Digital EEG data and analogue data, from E-prime, were collected via the MP150 system, with a sampling rate of 500 Hz, and were visualised real-time using AcqKnowledge 4.1 (Biopac Systems, Inc.).

For EEG data processing, data were first eye blink corrected and movement corrected (EOG), using automated ICA EOG correction in Acqknowledge 4.1 (Biopac Systems, Inc.), and then band pass filtered 0.1–30 Hz and Fourier transformed, using an in-house Matlab GUI, to accommodate differences in participant electrical brain activity conduction, that is, brain surface to recording electrode, relative (%) frequency bands power activity was extracted: delta (0.1–4.0 Hz), theta (4–7 Hz), alpha (7–14 Hz) and beta (15–30 Hz). For the purposes of the current study, we analyse delta/alpha frequency band activity only.

Clinical rating scales included the Positive and Negative Syndrome Scale (PANSS)^54^; Calgary Depression Scale for Schizophrenia^55^; Hamilton Rating Scale for Depression^56^; Young Mania Rating Scale^57^; Clinical Global Impression (CGI) scale^58^; Global Assessment of Functioning (GAF)^59^; and Simpson Angus Extrapyramidal Scale^60^. Chlorpromazine equivalents were calculated from current medication regimes^61^. Drug use history, nicotine, alcohol and methamphetamine were recorded using the Alcohol, Smoking and Substance Involvement Screening Test (ASSIST)^62^.

### Statistical analysis

**Fig. 1:**
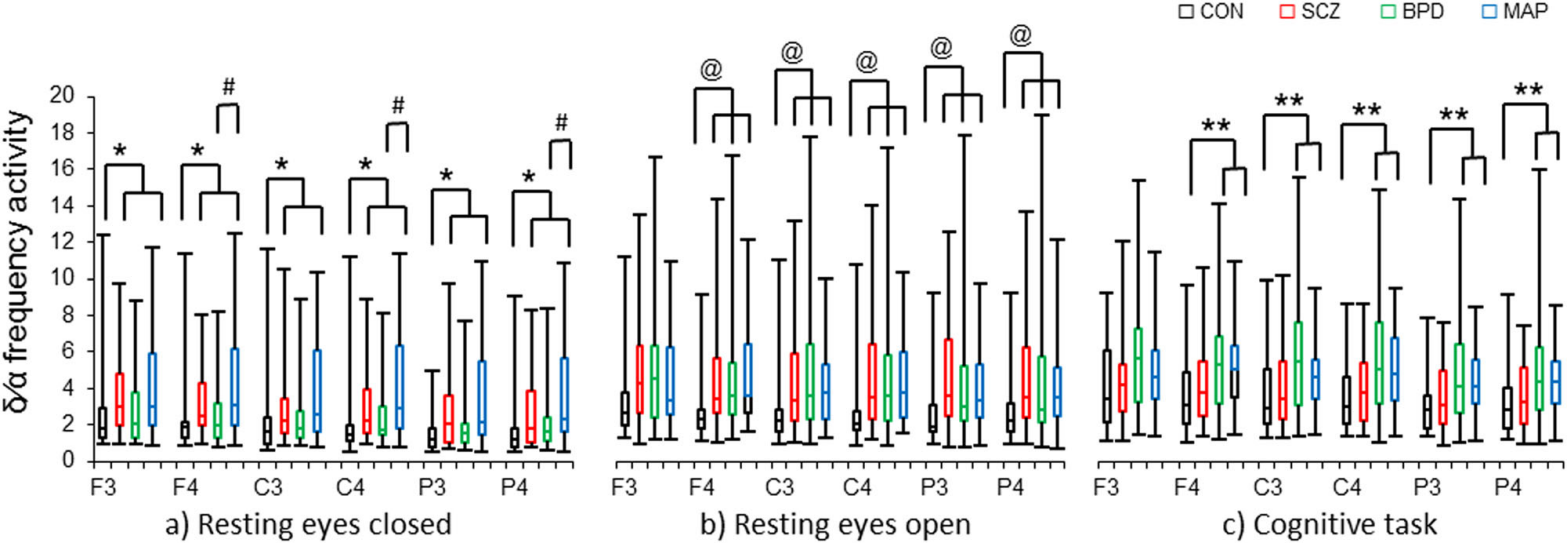
Delta/alpha frequency activity during three testing conditions: resting eyes closed (REC), resting eyes open (REO) and continuous performance task (CPT) in four groups: control (CON, *n* = 29), schizophrenia (SCZ, *n* = 28), bipolar I disorder with history of psychosis (BPD, *n* = 28) and methamphetamine-induced psychotic disorder (MPD, *n* = 24). **a** Delta/alpha frequency activity during REC was lower for CON than SCZ and MPD globally, and lower for BPD than MPD for right hemisphere electrodes (F_4_, C_4_, P_4_). **b** Delta/alpha frequency activity during REO was lower in CON than SCZ, BPD and MPD for all electrodes except left frontal (F_3_). **c** Delta/alpha frequency activity during the CPT was lower in CON than BPD and MPD for all electrodes except left frontal (F_3_). Median, interquartile and range are reported; *p* < 0.05

The data were largely non-parametric, as per Shapiro–Wilks distribution testing, and all EEG data parameters were skewed with significant group variance. There was an attempt to transform these data; however, no single transformation was held across delta/alpha frequency activity for the three testing conditions to address parametric bounds. There were three specific analysis approaches to determine group differences: (1) analysis across the four groups (CON, SCZ, BPD, MPD) (Table 1 and Fig. 1); (2) analysis across the three psychotic groups (SCZ, BPD, MPD) (Table 2); (3) medication analysis within the psychotic groups (Table 3). For the analysis of (1) and (2), first Kruskal–Wallis multivariate analysis of variances were performed, *p* < 0.05 (Fig. 1). Thereafter, correlational analyses, using Spearman’s rank order correlation, Rho >±0.6, *p* < 0.01, were applied across and within groups between variables that were reported in Tables 1 and 2 with their respective delta/alpha frequency activity data. To investigate the association of prescribed medications within the psychotic groups (3), Mann–Whitney *U* tests were applied and grouped by either *on* or *off* the respective medication being investigated. This included medications that were prescribed to at least 9 of the 80 participants with psychosis, refer to Table 2.

**Table 1:** Research participant groups ages, education, and behavioural performance

	Control (*n* = 29) 15 females/14 males		Schizophrenia (*n* = 28) 11 females/17 males		Bipolar I disorder (*n* = 28) 12 females/16 males		Methamphetamine-induced psychosis (*n* = 24) 10 females/14 males			
	Median	Range	Median	Range	Median	Range	Median	Range		
Age on day of imaging (years)	26	19–34^a^	29	20–39^a^	30.5	21–40	24.5	19–35	*H*_3,109_ = 12.76, *p* = 0.005	SCZ vs. CON&MPD*p* = 0.01; BPD vs. CON&MPD*p* = 0.01
Duration at school (years)	12	8–14	12	7–14	12	9–12^b^	10	7–12	*H*_3,109_ = 15.21, *p* = 0.001	MPD vs. CON*p* = 0.001; MPD vs. BPD*p* = 0.005
Tertiary education (years)	1	0–10	0	0–6	1.5	0–8^b^	0	0–4.5	*H*_3,109_ = 8.21, *p* = 0.04	MPD vs. CON*p* = 0.03; MPD vs. BPD*p* = 0.006
Total duration of education (years)	12	8–22	12	7–18	13.5	9–20^b^	10	7–16.5	*H*_3,109_ = 14.33, *p* = 0.02	MPD vs. CON*p* = 0.0009; MPD vs. BPD*p* = 0.0007
Handedness (left:right)	1:28		3:25		2:26		2:22			
*Continuous performance task*										
Correct responses (×/60)^c^	59	37–60	49	15–60^d^	58	45–60	50	16–60	*H*_3,105_ = 20.19, *p* = 0.002	CON vs. SCZ*p* < 0.001; CON vs. MPD*p* < 0.001; BPD vs. SCZp = 0.01
Overall response time (ms)^c^	209	103–532	438	57–1044^d^	302	203–371	275	105–676	*H*_3,105_ = 14.48, *p* = 0.002	CON vs. SCZ*p* < 0.001; CON vs. MPD*p* < 0.001; BPD vs. SCZp = 0.03
Errors of commission^c^	0	0–20	5	0–33	1	0–13	4	0–44	*H*_3,105_ = 9.70, *p* = 0.02	CON vs. SCZ*p* = 0.02; CON vs. MPD*p* = 0.003
Errors of omission	0	0–9^e^	3	0–45	0	0–18	0	0–21	*H*_3,105_ = 18.14, *p* < 0.001	SCZ vs. CON*p* < 0.001; SCZ vs. BPD*p* = 0.002; SCZ vs. MPD*p* = 0.03

^a^ SCZ and BPD older than CON and MPD

^b^ MPD lower education than CON and BPD

^c^ CON better behavioural performance than SCZ and MPD

^d^ BPD better performance than SCZ

^e^ SCZ greater number of omissions than all other groups, *p* < 0.05

*SCZ* schizophrenia, *BPD* bipolar disorder, *CON* controls, *MPD* methamphetamine-induced psychotic disorder

**Table 2:** Psychotic groups duration of illness, medications, and clinical scale scores

	Psychotic groups combined (*n* = 80)		Schizophrenia (*n* = 28)		Bipolar I disorder (*n* = 28)		Methamphetamine-induced psychosis (*n* = 28)			
	Median	Range	Median	Range	Median	Range	Median	Range		
Duration of psychotic disorder (years)	4.5	0.25–20.0	6.0	1.0–20.0	6.0	0.6–16.0^a^	1.0	0.25–13.0	*H*_2,80_ = 16.20, *p* = 0.003	MPD vs.SCZ*p* = 0.0007; MPD vs. BPD*p* = 0.0003
Chlorpromazine equivalents	113	0–1500	200	0–1500	88	0–600	113	0–450		
**Prescribed medications**										
*Any antipsychotic*	65		23		20		22			
First-generation antipsychotics	38		10		10^b^		18		*H*_2,80_ = 10.26, *p* = 0.005	MPD vs. SCZ*p* = 0.01; MPD vs. BPD*p* = 0.01
Haloperidol	24		3		6^b^		15		*H*_2,80_ = 17.78, *p* = 0.001	MPD vs. SCZ*p* = 0.001; MPD vs. BPD*p* = 0.01
Chlorporomazine	4		0		2		2			
Trifluoperazine	1		1		0		0			
Sulpiride	2		2		0		0			
Depot (flupentixol:zuclopenthixol:fluphenazine)	5:5:1		2:3:1		1:1:0		2:1:0			
Second-generation antipsychotics	33^c^		18		10		5		*H*_2,80_ = 10.48, *p* = 0.005	SCZ vs. MPD*p* = 0.007
Clozapine	10^c^		9		1		0		*H*_2,80_ = 15.15, *p* = 0.005	SCZ vs. MPD*p* = 0.04
Olanzapine	2		2		0		0			
Risperidone	15		5		5		5			
Quetiapine	6		2		4		0			
Moodstabilizers	35		6^d^		25		4		*H*_2,80_ = 35,95, *p* < 0.001	BPD vs. SCZ*p* < 0.0001; BPD vs. MPD*p* = 0.008
Lithium	14		1^d^		13		0		*H*_2,80_ = 24.76, *p* < 0.001	BPD vs. SCZ*p* = 0.006; BPD vs. MPD*p* = 0.004
Sodium valproate	24		5^d^		15		4		*H*_2,80_ = 11.26, *p* = 0.03	BPD vs. SCZ*p* = 0.03; BPD vs. MPD*p* = 0.02
Lamotrigine	2		0		2		0			
Serotonin/norepinephrine reuptake inhibitors										
Fluoxetine:citalopram:amitriptyline	5:1:2		2:1:1		2:0:0		1:0:1			
Anticholinergics—orphenadrine	16		5		2		9		*H*_2,80_ = 7.47, *p* = 0.02	MPD vs. BPD *tendencyp* = 0.05
**Clinical Scales**										
Positive and Negative Syndrome Scale										
Total score	43	30–100	48	30–100^e,f^	35	30–73	46	30–79	*H*_2,80_ = 13.5, *p* = 0.001	BPD vs. SCZ*p* = 0.009; BPD vs. MPD*p* = 0.003
Positive symptoms	8	7–30	12	7–24^e^	7	7–25	8	7–30	*H*_2,80_ = 8.47, *p* = 0.01	BPD vs. SCZ*p* = 0.006
Negative symptoms	11	7–30	13	7–30^e,f^	9	7–20	13	7–27	*H*_2,80_ = 24.89, *p* < 0.001	BPD vs. SCZp < 0.0001; BPD vs. MPD*p* < 0.0001
General psychopathology	22	16–50	23	16–50^e,f^	19	16–37	23	16–41	*H*_2,80_ = 6.99, *p* = 0.03	BPD vs. SCZ*p* = 0.01; BPD vs. MPD*p* = 0.03
Calgary Depression for Schizophrenia	1	0–13	2	0–8	0	0–13	1	0–9		
Hamilton Depression Rating Scale	3	0–16	3	0–15	1	0–16	3	0–12		
Young Mania Rating	1	0–20	2	0–10	0	0–20	2	0–5		
Clinical Global Impression of Illness Severity	2	1–4	3	1–4^e^	2	1–4	2	1–4	*H*_2,80_ = 10.40, *p* = 0.005	BPD vs. SCZ*p* = 0.003
Global Assessment of Functioning Scale	65	5–90	60	30–85^e^	71	5–85	65	31–90	*H*_2,80_ = 7.09, *p* = 0.02	BPD vs. SCZ*p* = 0.01
Simpson Angus Scale for Parkinsonism	0	0–16	1	0–16^e,f^	0	0–1	0	0–9	*H*_2,80_ = 15.16, *p* < 0.001	BPD vs. SCZ*p* = 0.001; BPD vs. MPD*p* = 0.04

^a^MPD shorter duration of psychotic disorder compared to SCZ and BPD

^b^MPD greater prescription of first-generation antipsychotics and haloperidol than BPD and SCZ

^c^SCZ greater prescription of second-generation antipsychotics and clozapine than MPD

^d^BPD greater prescription of moodstabilizers, lithium, and sodium valproate compare to SCZ and MPD

^e^BPD lower scores than SCZ

^f^BPD lower scores than MPD, *p* < 0.05

*SCZ* schizophrenia, *BPD* bipolar disorder, *MPD* methamphetamine-induced psychotic disorder

**Table 3:** Delta/alpha frequency activity association with second-generation antipsychotics during the continuous performance task

	Second-generation antipsychotic (*n* = 33)				No second-generation antipsychotic (*n* = 47)			*z*-score	*p*value
	Median	Min	Max		Median	Min	Max		
*Frontal*									
Left (F_3_)	3.37	1.15	12.06	**<**	5.03	1.42	15.36	2.5	0.01
Right (F_4_)	3.71	1.24	10.6	**<**	5.15	1.46	14.1	2.48	0.01
*Central*									
Left (C_3_)	3.22	1.31	10.42	**<**	4.89	1.35	15.57	2.44	0.01
Right (C_4_)	3.33	1.03	10.15	**<**	4.96	1.22	14.87	2.59	0.009
*Parietal*									
Left (P_3_)	2.65	0.85	9.35	**<**	4.63	1.02	14.4	2.9	0.003
Right (P_4_)	3.05	0.93	9.93	**<**	4.67	0.94	15.98	2.82	0.004

## Results

One hundred and nine research participants participated in the study: 28 individuals with diagnosis of SCZ, 28 with a diagnosis of BPD, 24 individuals with diagnosis of MPD and 29 healthy CON. Participants with SCZ and BPD were older than CON and MPD; all participants were between the ages of 19–40 years old. MPD participants reported lower educational achievement than CON and BPD. Several behavioural performance differences were found: CON performance surpassed SCZ and MPD for the number of correct responses, response time and errors of commission and omission; BPD performance surpassed SCZ for number of correct responses and response time; and SCZ participants made more errors of omission than participants with BPD or MPD (Table 1).

Duration of illness and medication recorded from the psychotic groups showed the following differences: MPD had a shorter duration of illness than BPD and SCZ; first-generation antipsychotics were more prescribed in MPD than BPD and SCZ (reflected in haloperidol prescription); second-generation antipsychotics were more prescribed in SCZ than MPD (reflected in clozapine prescription); and mood stabilisers were more prescribed in BPD than SCZ and MPD (reflected in lithium and sodium valproate prescription). Anticholinergic prescription (orphenadrine) differed across groups, with a tendency to be more prescribed in MPD compared to BPD (Table 2). Clinical scale scores differed across the patient participant groups: BPD participants had lower scores on the PANSS and its subscales compared to SCZ; the only aspect of the PANSS for which MPD participants did not report a lower score than participants with BPD was for the positive symptoms subscale; BPD participants were found to have greater psychosocial and occupational function (GAF) and were mentally less ill (CGI) than SCZ participants; and BPD participants reported fewer extrapyramidal symptoms than participants with SCZ or MPD (Table 2).

### Delta/alpha frequency activity group differences

During REC differences were found for delta/alpha frequency activity across all electrodes: F_3_ (*H*_3,109_ = 10.36, *p* = 0.01); F_4_ (*H*_3,109_ = 13.38, *p* = 0.03); C_3_ (*H*_3,109_ = 9.43, *p* = 0.02); C_4_ (*H*_3,109_ = 12.81, *p* = 0.005); P_3_ (*H*_3,109_ = 11.29, *p* = 0.01); P_4_ (*H*_3,109_ = 14.04, *p* = 0.002). Where SCZ and MPD delta/alpha frequency activity was higher than CON (SCZ vs.CON: F_3_*p* = 0.009, F_4_*p* = 0.002, C_3_*p* = 0.03, C_4_*p* = 0.007, P_3_*p* = 0.02, P_4_*p* = 0.02; MPD vs.CON: F_3_*p* = 0.01, F_4_*p* = 0.003, C_3_*p* = 0.006, C_4_*p* = 0.002, P_3_*p* = 0.001, P_4_*p* = 0.0004). Then MPD delta/alpha frequency activity was higher compared to BPD for right hemisphere only (MPD vs.BPD: F_4_*p* = 0.03, C_4_*p* = 0.04, P_4_*p* = 0.02) (Fig. 1a).

During REO differences were found for delta/alpha frequency activity across all electrodes, except for F_3_: F_4_ (*H*_3,107_ = 9.83, *p* = 0.02); C_3_ (*H*_3,107_ = 9.54, *p* = 0.02); C_4_ (*H*_3,107_ = 10.48, *p* = 0.01); P_3_ (*H*_3,107_ = 10.21, *p* = 0.01); and P_4_ (*H*_3,107_ = 11.36, *p* = 0.009). Where SCZ, BPD and MPD delta/alpha frequency activity was higher than CON (SCZ vs. CON: F_4_*p* = 0.008, C_3_*p* = 0.02, C_4_*p* = 0.01, P_3_*p* = 0.01, P_4_*p* = 0.01; BPD vs. CON: F_4_*p* = 0.01, C_3_*p* = 0.008, C_4_*p* = 0.02, P_3_*p* = 0.01, P_4_*p* = 0.02; MPD vs. CON: F_4_*p* = 0.01, C_3_*p* = 0.01, C_4_*p* = 0.002, P_3_*p* = 0.005, P_4_*p* = 0.001) (Fig. 1b).

During the CPT difference were found for delta/alpha frequency activity across all electrodes, except for F_3_: F_4_ (*H*_3,108_ = 8.61, *p* = 0.03); C_3_ (*H*_3,108_ = 10.14, *p* = 0.01); C_4_ (*H*_3,108_ = 9.79, *p* = 0.02); P_3_ (*H*_3,108_ = 8.45, *p* = 0.03); P_4_ (*H*_3,108_ = 9.94, *p* = 0.01). BPD and MPD delta/alpha frequency activity was higher than CON (BPD vs. CON: F_4_*p* = 0.01, C_3_*p* = 0.005, C_4_*p* = 0.01, P_3_*p* = 0.02, P_4_*p* = 0.009; MPD vs. CON: F_4_*p* = 0.02, C_3_*p* = 0.02, C_4_*p* = 0.01, P_3_*p* = 0.007, P_4_*p* = 0.008) (Fig. 1c).

### Delta/alpha frequency activity correlates

No significant correlations were found for delta/alpha frequency activity with variables listed in Table 1, or within the psychotic group for duration of illness, chlorpromazine equivalent or clinical scale scores in Table 2.

### Delta/alpha frequency associations with medication

Relationships between delta/alpha frequency activity were evident during the CPT testing condition only. With second-generation antipsychotics delta/alpha frequency activity was lowered globally (Table 3). The only within-group, that is, a single psychotic disorder, relationship was found in SCZ for P_3_ where second-generation antipsychotics lowered delta/alpha frequency activity (SCZ; *z* = 2.27, *p* = 0.02; *on* = 2.39 (0.85–7.63) *off* = 4.89 (2.14–6.78). Then first-generation antipsychotics delta/alpha frequency activity was higher for left frontal electrode (F_3_; *z* = 1.98, *p* = 0.04; *on* = 5.33 (1.15–15.36) *off* = 3.88(1.60–8.70)). Then haloperidol, a first-generation antipsychotic, delta/alpha frequency activity, was higher for right central electrode (C_4_; *z* = −2.09, *p* = 0.03; *on* = 6.02 (1.22–9.49) *off* = 4.32 (1.03–14.87)).

## Discussion

Our main finding was delta/alpha frequency activity, that is, higher delta and lower alpha synchronisation during three testing conditions is able to delineate psychotic disorders (Fig. 1). Although medication status was associated with differences in delta/alpha frequency activity during the cognitive task, this did not attenuate the delineation of the psychotic disorders.

First, delta/alpha frequency activity during REC was higher in SCZ and MPD globally, when compared to CON, then lower for BPD than MPD for right hemisphere. Previous studies report increased delta synchronisation during REC for SCZ when compared with CON^11–20^ and alpha desynchronisation SCZ^13,14,17,18,20,23,33–39^ when compared with CON. In BPD increased delta synchronisation^4,23,24^ and decreased alpha synchronisation has previously been reported^4,23,40,41^, although not all data are consistent^25^. Then abstinent previously dependent methamphetamine users without psychosis have been shown to lack EEG complexity^63^, suggesting higher delta synchronisation during REC for MPD, while no differences in alpha activity were reported^64,65^. Then for MPD, our findings suggest that higher delta/alpha frequency activity is specific to psychosis, and not drug related. The hemispheric differences between BPD and MPD suggest an attenuated deficit over the right hemisphere in BPD, and support BPD as a potential hemispheric disorder^66,67^. During REC delta/alpha frequency activity delineates SCZ and MPD from CON.

Second, delta/alpha frequency activity during REO was higher in SCZ, BPD and MPD for all electrodes, except left frontal, when compared to CON. Compared to REC, limited studies have reported EEG frequency activity during REO in SCZ, those that have do report higher delta activity^4,11,68,69^, else a lack of difference^13^. In BPD, higher delta activity has been reported during REO^4,23,24^, but not consistently^25^. While alpha desynchronisation is reproducibly reported for SCZ^13,14,17,18,20,23,33–39^ and BPD^4,23,40,41^. As reported for REC, no previous research has investigated delta or alpha activity in MPD; however, as suggested for REO, higher delta/alpha activity may be specific to psychosis, and not drug related. Lack of difference in delta/alpha frequency activity for left frontal electrode needs further study. During REO delta/alpha frequency activity delineates SCZ, BPD and MPD from CON.

Third, delta/alpha frequency activity during the cognitive task was higher in BPD and MPD for all electrodes, except left frontal, when compared to CON, and associations were found with medication during the cognitive task. There is limited research on EEG frequency activity during cognitive activation in psychotic disorders. Delta synchronisation over frontal cortex has been associated with poor cognitive performance^70^, this was apparent for SCZ (Table 1); however, relative delta/alpha activity was not correlated with behavioural performance in the current study. A single study in BPD reported greater delta activity during cognitive activation in BPD^71^. Reduced alpha frequency activity has been associated with reduced cognitive resource availability^72^. Lack of difference in delta/alpha frequency activity for left frontal electrode compliments findings during REO; however, further study is required to understand this lack of difference. During CPT delta/alpha frequency activity delineates BPD and MPD from CON.

Although cross-sectional design does not allow conclusions about causality, second-generation antipsychotic medication which were prescribed equally across groups lowered delta/alpha frequency activity globally during the cognitive task only (Table 3). This carried over to SCZ, where left parietal cortex delta/alpha frequency was lower. Previous work in this area is inconsistent; second-generation antipsychotics have been reported to decrease^73^ or increase^10,74–77^ delta activity, to increase^73,78^ and decrease^10,75^ alpha activity. Then prescription of first-generation antipsychotics increased delta/alpha frequency activity for left frontal electrode, and then haloperidol, a first-generation antipsychotic, increased delta/alpha frequency activity for right central electrode. One study in SCZ found that haloperidol acutely decreased delta, and then after 28 days of treatment, a similar effect was seen in delta, then alpha increased^34^. A study in CON found that acute dosing of haloperidol increased delta activity^77^. We hypothesise that second-generation antipsychotics attenuate the disparity in delta/alpha frequency activity, especially during cognitive activation, that is, phasic vs. tonic cortical arousal. Further study is required to investigate potential causality and role of both first-generation and second-generation antipsychotics in delta/alpha frequency activity during cognitive activation.

The study has several limitations. First, MPD participants were younger and reported a shorter duration of illness (Tables 1 and 2). No relationships with age or duration of illness were found for delta/alpha frequency activity, suggesting age and duration of illness were not potential confounders. Studies in abstinent previously dependent methamphetamine users report reduced complexity of EEG activity, for example, increased delta with no change in alpha^64,65^. Then duration of psychotic illness has been associated with increased delta^12^ and to hold no association with delta^17,18^ or alpha activity^12^, and increased delta has also been shown to serve as a good predictor for developing psychosis^79–81^. With limited EEG research in MPD, further study is required to address the impact of age and duration of illness on delta/alpha frequency activity.

Second, drug use history was recorded using the ASSIST^62^, a tool used in primary health care. Correlation analyses were performed using the total scores for nicotine, methamphetamine and alcohol across the four groups and within each of the groups, and no relationships were found with delta/alpha frequency activity. In future studies, a more sensitive measure should be employed, such as the Kreek–McHugh–Schuluger–Kellogg scale, which better quantifies subjective substances of abuse, including duration of substance abstinence^82^. Several cautions should be noted with using EEG delta/alpha as a neurophysiological biomarker to delineate psychotic disorders. First, the current cohorts were comprised of stable outpatients, and the findings may not be generalisable to patients with active psychosis. Second, the sensitivity and specificity of findings here need to be validated in larger patient samples, including other psychotic^83,84^ and more common mental disorders^36,85–88^. Third, our study did not address the question of whether delta/alpha frequency activity represent an endophenotype that is also altered in first-degree family members^4,23,25,89,90^.

In conclusion, this is the first study to show the delineation of psychotic disorders using delta/alpha frequency activity (Fig. 1). These findings support the involvement of thalamo-cortical mechanisms in the psychotic disorders. If findings are replicated, delta/alpha frequency activity may provide a useful neurophysiological biomarker to delineate the psychotic disorders.
